# News and Events

**Published:** 1911-11-04

**Authors:** 


### News and Events.

The ninth annual dinner of the Incorporated Association of Hospital Officers will be held at the Holborn Restaurant on Saturday next, November 4, at 7 p.m. Colonel Sir John Young, C.V.O., will be the guest of the evening.

The second Congregation of the Bristol University took place on Friday evening last week in the Victoria Rooms. Mr. Stear opened the proceedings with an organ recital. The procession entered the hall at eight o'clock. Among those present were the Vice-Chancellor, Sir Isambard Owen, who presided, the Lord Mayor and Sheriff, the Pro-Vice-Chancellor, Professor Michell Clarke, the Pro-Chancellors, the Deans of the Faculties, the Council of the University, and members of the Corporation of Bristol. Honorary degrees were conferred on Sir William Ramsay, K.C.B., Professor Alfred Marshall, Principal Wertheimer, and Mr. A. P. Chattock. When the Vice-Chancellor had taken up his position the Dead March in "Saul" was played as a token to the memory of Mr. H. O. Wills, the late Chancellor. The admittance to degrees then took place. After the ceremony the students indulged in a "rag," which, owing to the rain, was much quieter than that of last year.

Among the more noteworthy features of the Scottish vital statistics of the year 1909 is the lowness of the four principal rates—the birth-rate, the marriage-rate, the death-rate and the infantile-mortality rate. The birth-rate, the death-rate, and the infantile-mortality rate are each the lowest on record; the marriage-rate is lower than all previous marriage-rates, those of 1886 and 1887 excepted. This (1909) is the first year in which the Scottish death-rate has fallen below 16 per 1,000, and the second year—the other being 1907—in which the Scottish birth-rate has fallen below 28 per 1,000. A satisfactory feature of the statistics of the year is the continued decline of the death-rate from tuberculosis of all forms and from phthisis, both these rates being the lowest yet recorded. (Cd. 5879, price 2s. 9d.)

